# Measurements of Airborne Influenza Virus in Aerosol Particles from Human Coughs

**DOI:** 10.1371/journal.pone.0015100

**Published:** 2010-11-30

**Authors:** William G. Lindsley, Francoise M. Blachere, Robert E. Thewlis, Abhishek Vishnu, Kristina A. Davis, Gang Cao, Jan E. Palmer, Karen E. Clark, Melanie A. Fisher, Rashida Khakoo, Donald H. Beezhold

**Affiliations:** 1 Allergy and Clinical Immunology Branch, Health Effects Laboratory Division, National Institute for Occupational Safety and Health, Morgantown, West Virginia, United States of America; 2 Department of Community Medicine, West Virginia University, Morgantown, West Virginia, United States of America; 3 Department of Medicine, West Virginia University, Morgantown, West Virginia, United States of America; 4 Well WVU Student Health, West Virginia University, Morgantown, West Virginia, United States of America; Johns Hopkins University, United States of America

## Abstract

Influenza is thought to be communicated from person to person by multiple pathways. However, the relative importance of different routes of influenza transmission is unclear. To better understand the potential for the airborne spread of influenza, we measured the amount and size of aerosol particles containing influenza virus that were produced by coughing. Subjects were recruited from patients presenting at a student health clinic with influenza-like symptoms. Nasopharyngeal swabs were collected from the volunteers and they were asked to cough three times into a spirometer. After each cough, the cough-generated aerosol was collected using a NIOSH two-stage bioaerosol cyclone sampler or an SKC BioSampler. The amount of influenza viral RNA contained in the samplers was analyzed using quantitative real-time reverse-transcription PCR (qPCR) targeting the matrix gene M1. For half of the subjects, viral plaque assays were performed on the nasopharyngeal swabs and cough aerosol samples to determine if viable virus was present. Fifty-eight subjects were tested, of whom 47 were positive for influenza virus by qPCR. Influenza viral RNA was detected in coughs from 38 of these subjects (81%). Thirty-five percent of the influenza RNA was contained in particles >4 µm in aerodynamic diameter, while 23% was in particles 1 to 4 µm and 42% in particles <1 µm. Viable influenza virus was detected in the cough aerosols from 2 of 21 subjects with influenza. These results show that coughing by influenza patients emits aerosol particles containing influenza virus and that much of the viral RNA is contained within particles in the respirable size range. The results support the idea that the airborne route may be a pathway for influenza transmission, especially in the immediate vicinity of an influenza patient. Further research is needed on the viability of airborne influenza viruses and the risk of transmission.

## Introduction

Influenza continues to be a major public health concern because of the substantial health burden from seasonal influenza and the potential for a severe pandemic. Although influenza is known to be transmitted by infectious secretions, these secretions can be transferred from person to person in many different ways, and the relative importance of the different pathways is not known. The likelihood of the airborne transmission of influenza virus by infectious aerosols is particularly unclear, with some investigators concluding that airborne transmission is a key route (reviewed in [1], [2], [3]), while others maintain that it rarely, if ever, occurs (reviewed in [4]). The question of airborne transmission is especially important in healthcare facilities, where influenza patients tend to congregate during influenza season, because it directly impacts the infection control and personal protective measures that should be taken by healthcare workers. During the 2009 H1N1 pandemic, for example, a United States Institute of Medicine (IOM) panel recommended that healthcare workers in close contact with influenza patients wear respirators to avoid infectious aerosols [5]. This recommendation was subsequently adopted by some health authorities such as the US Centers for Disease Control and Prevention (CDC), but not by others, such as the World Health Organization (WHO). The IOM panel also noted that many questions about the airborne transmission of influenza are unresolved, and the issue remains controversial.

The probability of the airborne transmission of influenza virus depends in part on the amount of aerosolized virus to which people are exposed. Two recent studies have measured the amount of airborne influenza viral RNA in healthcare facilities during the influenza season [6], [7]. Both studies found that the highest concentrations of influenza RNA were detected in locations where, and during times when, the number of influenza patients was highest. The studies also found that 42 to 53% of the influenza viral RNA was contained in airborne particles less than 4 µm in aerodynamic diameter (the respirable size fraction). Aerosol particles in this size range are of particular concern because they can remain airborne for an extended time and because they can be drawn down into the alveolar region of the lungs during inhalation. The infectious dose required for inoculation by the aerosol route relative to contact or droplet transmission is unclear, but two reviews of previous studies concluded that the infectious dose by the aerosol route is likely considerably lower than the infectious dose by intranasal inoculation [2], [8], and that aerosol inoculation results in more severe symptoms [8], presumably because aerosol particles are able to deposit deeper in the respiratory tract. However, the viability of influenza viruses in particles of different sizes and the persistence of viable airborne virus in the environment are not yet known.

A few studies have examined airborne influenza virus production at the source (influenza patients). Fabian et al. [9] and Stelzer-Braid et al. [10] detected influenza viral RNA produced by influenza patients during breathing and talking. Fabian et al. [9] showed that 60% of patients with influenza A and 14% of patients with influenza B had detectable levels of viral RNA in their exhaled breath; they also reported that over 87% of the exhaled particles were less than 1 µm in diameter. Milton et al. [11] collected aerosol particles exhaled by influenza patients and found that patients shed about 33 viral copies/minute in aerosol particles ≥5 µm and 187 viral copies/minute in particles <5 µm. They also showed that surgical masks substantially reduced particle release (especially for large particles), and found culturable virus in the breath from two subjects. Despite these studies, however, little is known about the production of potentially infectious aerosols by influenza patients.

The purpose of this study was to measure the amount and size of airborne particles containing influenza virus that are produced by patients when they cough. A better understanding of the amount of potentially infectious material released by patients and the size of the particles carrying the virus will assist in determining the possible role of airborne transmission in the spread of influenza and in devising measures to prevent it.

## Results

**Figure 1:**
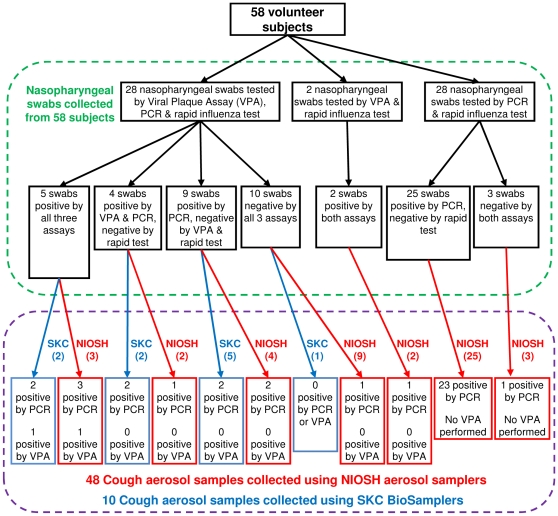
Flow chart showing patients and tests performed. The numbers in parentheses are the number of aerosol samples collected using SKC BioSamplers and NIOSH aerosol samplers.

Fifty-eight volunteer subjects (38 male, 20 female, ages 18 to 33) participated in the study during October-November 2009, when the pandemic influenza A (H1N1) virus predominated. Seven subjects reported receiving a seasonal influenza vaccination; none had received a vaccination against the 2009 H1N1 influenza virus. At the clinic, the rapid influenza tests from 7 subjects were positive. In subsequent testing, influenza viral RNA was detected by quantitative real-time reverse-transcription PCR (qPCR) in nasopharyngeal swabs from 43 of 56 subjects, with a median viral copy number of 51 per sample (SD 161 after the exclusion of one outlying value of 3727), where each sample consisted of two swabs collected from each patient. Viral plaque assays (VPA) were performed on nasopharyngeal swabs from 30 subjects. Viable influenza virus was detected in 11 of these VPA's (median 6.0×10^4^ pfu/ml, SD 2.85×10^5^). Nasopharyngeal swabs from two subjects were not tested using qPCR but were positive by rapid test and VPA. For two other subjects, influenza virus was not detected in the nasopharyngeal swabs by qPCR or rapid test, but was subsequently detected in their cough aerosols by qPCR. Overall, influenza virus was detected in 47 of the 58 subjects. A flowchart showing the subjects and the results of the tests performed to detect influenza is presented in Figure 1.

**Figure 2:**
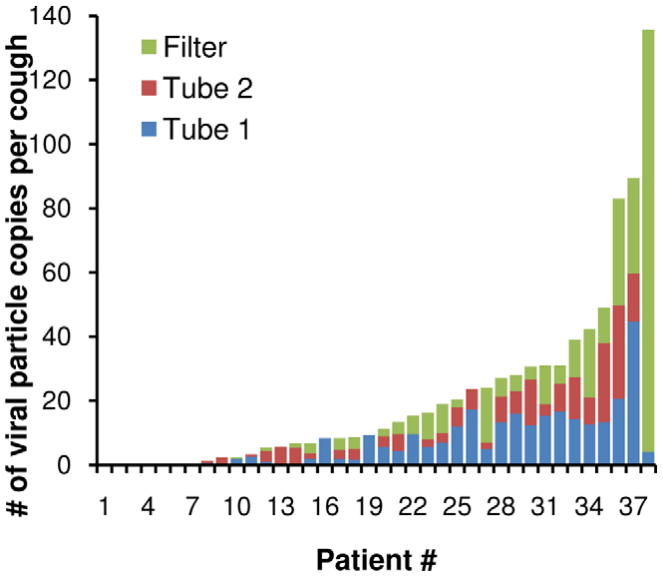
Influenza viral RNA collected from coughs using the NIOSH two-stage aerosol sampler. Influenza viral RNA was detected in the cough aerosols from 32 of 38 influenza-positive patients. This plot shows the number of viral copies per cough detected in aerosol particles collected in sampler tube 1 (>4 µm), tube 2 (1 to 4 µm) and the filter (<1 µm) for each patient, ordered from minimum to maximum. The particles collected in tube 2 and on the filter are respirable (able to reach the alveolar region).

Because results from the VPA and qPCR assays were not available until several days after patients were tested, cough-generated aerosols were collected from all patients and their influenza status was determined afterwards. Cough-generated aerosols were collected from 48 subjects using the NIOSH two-stage aerosol sampler, 38 of whom were later found to be influenza-positive as described above. Influenza viral RNA was detected in at least one sampler stage for 32 of the 38 influenza-positive subjects. The amount of influenza viral RNA expelled by each patient during coughing is shown in Figure 2, while the median number of viral particle copies per cough and the distribution of the viral RNA by particle size for these subjects are shown in Table 1. Sixty-five percent of the influenza viral RNA was found in particles less than 4 µm in diameter. VPA's were performed on cough aerosols collected from 20 subjects using the NIOSH sampler, 12 of whom were influenza-positive. One of these cough aerosol VPA's was positive, with 0.8 pfu/ml per cough found in the first tube. This sample was from the patient with the third-highest cough aerosol viral particle count by qPCR (83 viral copies per cough). A summary of the VPA results for the NIOSH and SKC samplers is shown in Table 2.

**Table 1:** Influenza viral RNA detected in the NIOSH two-stage aerosol sampler.

*Aerosol particle size range (aerodynamic diameter)*	*Median # of viral copies per cough*	*% of viral RNA contained in particles in this size range*	*% of subjects whose cough aerosol contained viral RNA-laden particles in this size range*
>4 µm	6.3 (SD 9.0)	35%	90%
1 to 4 µm	3.3 (SD 6.9)	23%	81%
<1 µm	3.7 (SD 23.7)	42%	75%
All particles	15.8 (SD 29.3)	100%	100%

The NIOSH two-stage sampler was used to collect cough aerosols from 48 subjects, 38 of whom were later confirmed to have influenza. Influenza viral RNA was detected in at least one sampler stage for 32 of the viral positive subjects (84%). This table shows the viral copy number and distribution of particle sizes for the 32 subjects for whom influenza viral RNA was detected in their cough-generated aerosol particles. As illustrated by the large standard deviations (SD), the amount of influenza viral RNA in the cough aerosols varied tremendously between patients.

**Table 2:** Viral plaque assay results for cough-generated aerosols.

*Aerosol sampler*	*Total # of subjects for whom VPA was performed on cough aerosol*	*Total # of these subjects who were influenza-positive (by qPCR or VPA)*	*# of influenza-positive nasal swabs*		*# of influenza-positive cough aerosols*	
			*qPCR*	*VPA*	*qPCR*	*VPA*
NIOSH two-stage sampler	20	12	9 (of 18)	7	8	1
SKC BioSampler	10	9	9	4	6	1

Nasopharyngeal swabs and cough aerosol samples from 30 subjects were cultured for viable influenza virus. This table shows the number of samples found to be influenza-positive by qPCR and VPA.

The SKC BioSampler was used to collect cough aerosols from 10 subjects, 9 of whom were influenza-positive. Influenza viral RNA was detected in 6 of the cough aerosol samples by qPCR (median copy number per cough 30, SD 70, excluding 1 sample with 355 viral copies per cough). Viable influenza virus was found in 1 of these cough aerosol samples; 5 pfu/ml per cough were detected in this sample by VPA and 10 viral particles per cough were detected using qPCR.

The oral temperature, self-reported symptoms, cough volumes, and peak cough flow rates of all subjects are shown in Table 3. Influenza-positive subjects reported more symptoms overall than influenza-negative subjects, but there was no clear relationship between any of the clinical parameters and the amount of influenza RNA contained in the cough-generated aerosols. The complete set of experimental data for this paper is available online as supplemental material (Results S1).

**Table 3:** Clinical presentation of study participants.

	*Influenza-positive*	*Influenza-negative*
# of subjects	47	11
Oral temperature (°C)	37.4 (SD 0.7)	37.1 (SD 0.4)
Days of symptoms (median)	2 (SD 5)	4 (SD 4)
% of subjects reporting:		
Fever/chills	81%	27%
Headache	81%	45%
Fatigue	74%	45%
Cough	85%	55%
Sore throat	87%	18%
Muscle aches	77%	36%
Cough volume (liters)	2.7 (SD 1.1)	3.1 (SD 1.3)
Cough peak expiratory flow rate (liters/minute)	426 (SD 163)	454 (SD 194)

Average values are given, except for days of symptoms, for which the median is shown.

## Discussion

The production and release of potentially infectious aerosol particles by influenza patients is a major concern because of the possibility that these particles could transmit the disease to healthcare providers and to other patients. However, little is known about the amount, size distribution and viability of the influenza virus-laden particles generated by these patients when they cough. Our study found that 81% of the influenza-positive patients had detectable levels of influenza viral RNA in their cough aerosols. Further, 65% of the influenza viral RNA was contained in particles in the respirable size fraction (<4 µm). These are particles that are small enough to remain airborne for an extended time and to be inhaled into the alveolar region of the lungs. Particles in this size fraction are of particular concern because some human experiments have suggested that a much smaller dose of influenza virus is needed to initiate an infection when it is deposited in the alveolar region compared to intranasal inoculation [8]. It is interesting to note that the fraction of influenza RNA-laden particles in the respirable size range was somewhat higher in this study than the 42 to 53% that was found during aerosol sampling in healthcare facilities [6], [7]. This may in part reflect a loss of large particles in the cough aerosol collection system, and may also be due to the natural coagulation of aerosol particles that occurs over time.

A comparison of the amount of influenza viral RNA in the nasopharyngeal swabs and the cough-generated aerosols found a correlation coefficient of r = 0.73 for the NIOSH sampler and 0.85 for the SKC BioSampler (in both cases, excluding one outlying point). This suggests that, as might be expected, patients with higher viral loads in their nasopharyngeal region generally shed more viral RNA during coughing. It is especially interesting to note that the amount of viral RNA detected in cough aerosols varied widely from patient to patient; in fact, 45% of the influenza viral RNA from cough aerosols collected using the NIOSH sampler came from just 4 of 38 subjects with influenza. Large variations in the amount of virus in cough aerosols are not surprising, since influenza viral shedding varies significantly from patient to patient and over the course of the illness [12], and since previous studies have shown that some individuals shed much greater quantities of aerosol particles during breathing and coughing than do others [13]. However, this does suggest that some influenza patients might be able to serve as “superspreaders” who are considerably more likely than other patients to transmit the disease by the airborne route [14].

We were able to show that viable influenza virus was present in the cough-generated aerosols from 2 of 11 subjects for whom viable virus was found in their nasopharyngeal swabs. This demonstrates that, at least in some cases, influenza patients do release airborne particles containing potentially infectious virus. This result supports the theory that airborne transmission of influenza is possible, although additional factors such as the survival time and infectivity of the airborne virus remain unclear. It is important to note, however, that our results are almost certainly an underestimate of the amount of viable airborne virus that is released. It is known that the process of collecting aerosols frequently leads to inactivation of viruses [15], [16]. The NIOSH two-stage sampler collects particles in dry tubes and on a filter, which can damage delicate viruses by desiccation or mechanical damage. The SKC BioSampler collects particles in liquid, which helps preserve viability, but it does not collect small particles efficiently and is not size-selective. In laboratory experiments collecting aerosolized influenza virus, the viability of virus collected with an SKC BioSampler was about 4 times higher than the viability of virus collected with the NIOSH sampler [17]. In addition, viral plaque assays are widely used to study virus viability, but they are not sensitive enough to detect small quantities of virus. Better aerosol collection methods and more sensitive viability assays may lead to higher estimates of the amount of viable airborne virus released by people with influenza.

Finally, two limitations in our study should be noted. First, studies of cough-generated aerosols have shown that human coughs produce a tremendous range of particle sizes, ranging from less than 100 nm to visible drops larger than a millimeter [18], [19], [20], [21]. Large aerosol particles settle much more quickly than do small particles, and are also more likely to impact and stick to surfaces. Our cough aerosol collection system collects small particles, but many larger particles are almost certainly lost. Thus, the collection system should provide a good representation of the amount of virus contained in aerosol particles capable of remaining airborne for several minutes or longer, but it does not collect the total amount of viral material expelled by a coughing patient. Second, all of the patients who participated in our study were young otherwise healthy adults who were ambulatory and able to be treated in an outpatient clinic. Patients who are more severely ill would be expected to have higher viral loads and more respiratory fluids in the lungs [22], which could increase the amount of virus in the cough-generated aerosols. In addition, influenza virus shedding peaks early in the course of the illness (typically about 2 days after the onset of symptoms [12]). In our study, 40% of subjects with influenza reported that 3 days or more had passed since the onset of their symptoms. Thus, many had likely passed the peak of viral shedding by the time they entered the study.

In conclusion, our study measured the amount and size distribution of aerosol particles containing influenza viral RNA that were produced by influenza patients as they coughed. Our results show that influenza patients do produce aerosol particles containing measurable amounts of influenza virus while coughing. Further, much of the viral RNA is contained within particles that can remain airborne for an extended time and that can enter the alveolar region of the lungs if they are inhaled. Our study was also able to demonstrate that at least some influenza patients expelled airborne particles containing viable influenza virus. Our results support the idea that airborne transmission may play a role in the spread of influenza, especially in the immediate vicinity of an influenza patient.

## Materials and Methods

### Ethics Statement

All procedures involving human subjects were reviewed and approved by the National Institute for Occupational Safety and Health (NIOSH) and West Virginia University (WVU) Institutional Review Boards. Written informed consent was obtained from all study participants.

Volunteer subjects were recruited from patients presenting with influenza-like symptoms at the student health clinic of WVU in Morgantown, West Virginia, USA, during October and November of 2009. After providing informed consent, each subject was given a rapid influenza test (QuickVue Influenza A+B test, Quidel). The rapid test was used to provide an initial estimate of influenza case numbers; however, because the sensitivity of the test was reported to be low [23], subjects were allowed to continue participating in the study regardless of the outcome. Two nasopharyngeal mucus swabs were collected for analysis by qPCR and viral plaque assay (VPA), the subject's oral temperature was taken, and the subject was asked to answer a brief health questionnaire.

**Figure 3:**
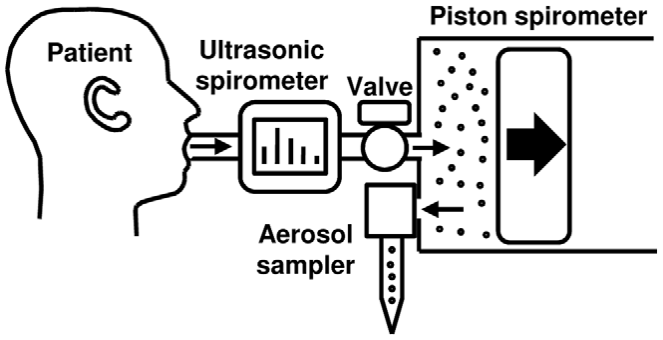
Cough aerosol particle collection system. Before each test, the piston spirometer was purged and partially filled with 5 liters of clean dry air. When the patient coughed into the system mouthpiece, the cough flowed through an ultrasonic spirometer which measured the cough volume and flow rate. The cough then flowed through a valve and into the piston spirometer, displacing the piston to the right. When the subject finished coughing, the valve was closed and the aerosol sampler was turned on. The cough aerosol was pulled out of the spirometer and collected by the aerosol sampler. As the aerosol sampler drew air, the piston moved to the left until no air remained in the spirometer.

Cough-generated aerosols from the volunteer subjects were collected using the cough aerosol particle collection system (Figure 3) similar to that described previously [24]. The system consisted of an ultrasonic spirometer (Easy One, NDD Medical Technologies) and a 10 liter piston-style spirometer (SensorMedics model 762609) modified to allow aerosol collection using a NIOSH two-stage cyclone aerosol sampler [6] or an SKC BioSampler with a 5 ml collection vessel (#225-9593, SKC). The NIOSH sampler collected cough aerosol particles in a 15 ml centrifuge tube (stage 1; #35-2096, Falcon), a 1.5 ml centrifuge tube (stage 2; #02-681-339, Fisher Scientific) and a 37 mm polytetrafluoroethylene (PTFE) filter with 2 µm pores (#225-27-07, SKC). The NIOSH sampler conforms to the ACGIH/ISO criteria for respirable particle sampling [25]. The flow rate through each NIOSH sampler was set to 3.5 liters/minute with a flow calibrator (Model 4143, TSI) before use. The SKC BioSampler collects aerosols into 5 ml of universal transport media (UTM; Copan Diagnostics) at 12.5 liters/minute.

Before each collection, the system was purged and the piston spirometer was partially filled with 5 liters of clean dry air. The subject was then asked to sit in front of the system, inhale, exhale, inhale as deeply as possible, seal their mouth around the mouthpiece, and cough into the machine using as much of the air in their lungs as possible. After each cough, the system valve was closed and the cough-generated aerosol was collected using the aerosol sampler. This procedure was repeated twice for a total of three coughs from each subject.

After collection, the nasopharyngeal swabs were immersed in 1 ml UTM in a storage tube. For the NIOSH samplers, 1 ml of UTM was added to each sampler tube, while the sampler filters were immersed in 1 ml UTM in a 50 ml centrifuge tube. For the SKC sampler, the UTM collection media was removed from the sampler and placed a storage tube. All tubes were vortexed thoroughly. 500 µl of UTM was then drawn from each tube and mixed with 500 µl of Lysis/Binding Solution Concentrate (LBSC; Ambion) in fresh tubes. The tubes with the remaining UTM were stored overnight at 4°C, while the tubes with UTM and LBSC were stored overnight at −20°C. In some cases, UTM was not used; instead, 500 µl of LBSC was added directly to each tube, and the tubes were stored overnight at −20°C.

To extract the sample RNA, tubes containing samples in LBSC were thawed, carrier RNA (Ambion) was added to enhance RNA extraction and XenoRNA (Applied Biosystems) was added as a qPCR internal control. Total RNA was extracted as previously reported [6] and immediately transcribed into cDNA using High Capacity RNA to cDNA Master Mix (Applied Biosystems).

Real-time quantitative PCR was performed with a Model 7500 Fast Real-Time PCR system (Applied Biosystems) using influenza A matrix-specific primers and probe (Spackman, 2002).

To determine the relative genome copy from seasonal influenza A-positive aerosol samples, a standard curve was generated from 10-fold serial dilutions of the influenza M1 matrix gene and analyzed alongside all qPCR reactions. All reactions were run in duplicate. A negative control without template was included in all real-time PCR reactions. Real-time PCR detection of the XenoRNA internal control was performed using the XenoRNA Control TaqMan Gene Expression Assay from the TaqMan Cells to C_t_ Control Kit (Applied Biosystems). The internal controls were amplified in all samples.

For the viral plaque assay (VPA), Madin Darby canine kidney (MDCK) cells (CCL-34) were purchased from the American Type Culture Collection (ATCC, Manassas, VA). Cells were propagated and maintained in 75-cm^2^ flasks (Corning CellBind Surface, Corning, NY). Growth medium for MDCK cells consisted of Eagle's minimal essential medium (EMEM, ATCC) supplemented with 10% fetal bovine serum (Hyclone Laboratories, Inc, Logan, Utah), 0.4 units/ml penicillin (Invitrogen, Carlsbad, CA), and 0.4 µg/ml streptomycin (Invitrogen). Cells were incubated at 35°C in a humidified 5% CO_2_ incubator until about 90% confluent. The VPA was performed by trypsinizing, washing and plating MDCK cells at a density of 2.0×10^6^ per well (CoStar 6-well tissue culture plate, Corning). Cells were incubated at 35°C in a humidified 5% CO_2_ incubator overnight. Confluent cellular monolayers were next washed two times with PBS (Invitrogen) and treated with the clinical samples. Following 45 min of adsorption, virus-infected MDCK cells were washed with phosphate buffered saline (PBS, Gibco), overlaid with an agarose medium solution and incubated at 35°C in a humidified 5% CO_2_ incubator for 48 h. Plaques were visually enumerated and plaque forming units (PFU)/ml were calculated.

Initially, VPA's were performed only on nasopharyngeal swabs and cough aerosols from subjects with positive rapid influenza tests. After a few days, our preliminary results indicated that the rapid tests had a lower-than-expected sensitivity, and we changed our methodology to perform VPA's on all samples.

## Supporting Information

Results S1 Complete set of experimental results for this study.
(TXT)
